# Assessment of valve implantation in the descending aorta as an alternative for aortic regurgitation patients not treatable with conventional procedures

**DOI:** 10.1007/s10237-022-01665-3

**Published:** 2022-12-23

**Authors:** A. García-Galindo, R. Agujetas, J. R. López-Mínguez, C. Ferrera

**Affiliations:** 1 Departamento de Ingeniería Mecánica, Energética y de los Materiales and Instituto de Computación Científica Avanzada (ICCAEx), Universidad de Extremadura, E-06006 Badajoz, Spain; 2 Sección de Cardiologıa Intervencionista, Servicio de Cardiologıa, Hospital Universitario de Badajoz, Avda. de Elvas s/n, E-06006 Badajoz, Spain

**Keywords:** Computational fluid dynamics, Fluid-structure interaction, Aortic regurgitation, Descending aorta, Bileaflet mechanical heart valve

## Abstract

**Background:**

Aortic Regurgitation (AR) produces the entrance of an abnormal amount of blood in the left ventricle. This disease is responsible for high morbidity and mortality worldwide and may be caused by an aortic valve dysfunction. Surgical and transcatheter aortic valve replacement (TAVR) are the current options for treating AR. They have replaced older procedures such as Hufnagel’s one. However, some physicians have reconsidered this procedure as a less aggressive alternative for patients not eligible for surgical or TAVR. Although Hufnagel suggested a 75% regurgitation reduction when a valve is placed in the descending aorta, a quantification of this value has not been reported.

**Methods:**

In this paper, CFD/FSI numerical simulation is conducted on an idealized geometry. We quantify the effect of placing a bileaflet mechanical heart valve in the descending aorta on a moderate-severe AR case. A three-element Windkessel model is employed to prescribe pressure outlet boundary conditions. We calculate the resulting flow rates and pressures at the aorta and first-generation vessels. Moreover, we evaluate several indices to assess the improvement due to the valve introduction.

**Results and conclusions:**

Regurgitation fraction (RF) is reduced from 37.5% (without valve) to 18.0% (with valve) in a single cardiac cycle. This reduction clearly shows the remarkable efficacy of the rescued technique. It will further ameliorate the left ventricle function in the long-term. Moreover, the calculations show that the implantation in that location introduces fewer incompatibilities’ risks than a conventional one. The proposed methodology can be extended to any particular conditions (pressure waveforms/geometry) and is designed to assess usual clinical parameters employed by physicians.

## Introduction

Heart Failure (HF) affects 6 million Americans, and this number is expected to reach 8 million by 2030 (HF total costs projection is $69.8 billion) (Virani et al. 2021). One of the diseases that can lead to HF is chronic Aortic Regurgitation (AR). In AR, there is an entrance of an abnormal amount of blood in the left ventricle (LV) coming from the aorta. It mainly affects people older than sixty years (Singh et al. 1999). Its prevalence is 4.9% of the American population and 0.5% for moderate, severe, and acute cases (Singh et al. 1999; Maurer 2006). The incidence increases in the Western World as the population is aging (Cheng et al. 2021; Stachon et al. 2020; Goldsweig et al. 2019).

AR may be caused by an aortic valve (AV) dysfunction (Fernández-Golfín 2020). Its treatment is based on either a valve replacement or medical care. As the yearly death rate ranges from 10% to 20% (El-Gamel 2021) in medically treated AR patients, surgical aortic valve replacement (SAVR) has traditionally been considered the gold standard (Franzone et al. 2016). For those AR patients suffering from Aortic Stenosis (AS), SAVR results in an 80% survival rate compared to a 20% for medical treatment (Carabello 2008). Transcatheter aortic valve replacement (TAVR) is a minimally invasive alternative approved by the United States Food and Drug Administration in 2011 (Goldsweig et al. 2019; Praz et al. 2015). It is widely used in most of the patients over 75 years old with severe AS (Praz et al. 2015).

However, TAVR is excluded or constitutes a technical challenge for specific AR cases: bicuspid AV (Bellini et al. 2021), large aortic annulus diameter ($> 30$ mm), ascending aorta or aortic root dilation, ascending aorta aneurysms, or lack of calcification in the AV (Stachon et al. 2020; El-Gamel 2021). The calcium absence or an excessive diameter of the annulus in the aortic root dilatation complicate the valve anchorage. Although new generation devices can achieve good postoperative results, the risk of valvular migration and malpositioning are considerable (El-Gamel 2021; Markham et al. 2020). Therefore, TAVR is an off-label indication for specific symptomatic pure AR (PAR) patients (without AS) (Stachon et al. 2020; Goldsweig et al. 2019; El-Gamel 2021; Arias et al. 2019). New devices need to be developed for such patients (El-Gamel 2021; Markham et al. 2020), and SAVR remains the preferred option.

There are several operations for repairing the aortic root and ascending aorta (David and Feindel 1992; Sarsam and Yacoub 1993; Ross 1967; Bentall and De Bono 1968). These operations involve multiple procedures that require 3D geometrical thinking, manual skills, and a vast experience (Miller 2003). Although these procedures continue evolving (Nezafati et al. 2015), many patients are not eligible for them. Age, cardiac comorbidities, active endocarditis or a history of previous surgeries further complicate those procedures (Szeto et al. 2007; McKellar and Sundt 2009; Dhurandhar et al. 2016). Some authors point doubts about the treatment of an AV malfunction due to aneurysms (Sheick-Yousif et al. 2008), and there are limited data regarding aortic root replacements after a first cardiac surgery (Heubner et al. 2019). Therefore, less invasive (Heubner et al. 2019) or alternate procedures are recommended for those patients.

Recently, a group of surgeons has proposed recovering Hufnagel’s procedure (Rose et al. 1954; Hufnagel et al. 1958) to treat these patients (Fantidis et al. 2014). Hufnagel implanted a prosthesis in the descending aorta, immediately distal to the left subclavian artery, to treat aortic insufficiency in the early 50’s (Rose et al. 1954). He reported a heart size reduction (Leitz and Ziemer 2017) and no cases of thrombosis or degeneration (De Martino et al. 2020). Nevertheless, the human data retrieved was limited between 1952 and 1960: 4000 prostheses were distributed, the number of implantations was unknown, and only 55 patients provided data (26 deaths were not valve related in that sample) (van Herwerden and Serruys 2002). This technique was abandoned in favor of orthotopic position implantation when the cardiopulmonary bypass was developed (Vendramin et al. 2022). Since then, it has only been employed in 4 humans with a malfunction of biological prostheses (Cale et al. 1993), in a dog (Arai et al. 2007), and as a temporary compassionate treatment in a patient with a complicated surgical history (Fukuhara et al. 2020). Fukuhara et al. (2020) employed a modified Hufnagel procedure by performing a percutaneous implantation as suggested by Boudjemline and Bonhoeffer (2002) after confirmation in lambs. Hufnagel et al. (Rose et al. 1954) estimated a 75% regurgitant flow reduction by implanting this prosthesis. This estimation is based on the regurgitant flow percentage that can be controlled at that location (Hufnagel and Gomes 1976). Nevertheless, although cited in several papers, this quantity has neither been calculated nor confirmed (Fishbein and Roberts 1975). This data is crucial for surgeons or interventional cardiologists before an intervention, and it could be obtained by employing Computational Fluid Dynamics (CFD).

CFD has been employed to assess some cardiovascular diseases (Ueda et al. 2018; Kojima et al. 2021). This assessment can be personalized, non-invasive, and cost-reduced (Bonfanti et al. 2019; Swanson et al. 2020). Its combination with Fluid-Structure Interaction (FSI) analysis allows heart valves , artery wall dynamics evaluation, or the biomechanical response to an LV assist device (Yoganathan et al. 2004; Dumont et al. 2007; Gao and Zhang 2020; Kasinpila et al. 2021). Regarding AR, CFD/FSI has been applied to analyze the effect on the hemodynamics of different AV morphological features (aortic sinuses (Pan et al. 2015; Kivi et al. 2020), coronary ostia (Youssefi et al. 2017), bicuspid valves (Berdajs et al. 2018; Lavon et al. 2018)). It can predict the AR degree in different anatomies and assist physicians in choosing the valve size and the optimal deployment location (De Jaegere et al. 2016; Luraghi et al. 2019). Long-term effects associated with the implantation can also be numerically predicted, such as thrombus risk (Bianchi et al. 2019) or the outcome of implantation in bicuspid AVs (Dowling 2019; Dowling et al. 2021).

For all these reasons, in this paper, we have employed CFD/FSI to analyze the effect of placing a bileaflet mechanical heart valve in the descending aorta on a moderate-severe AR case (Bolen et al. 2011) according to Doppler Echocardiography (DE) severity grading assessment (Solomon 2007). We have applied Hufnagel’s technique (Rose et al. 1954), as recently proposed (Fantidis et al. 2014), and have obtained the regurgitant flow reduction, the stroke volume, and the regurgitant fraction. Also, we have calculated different indices that consider the hemodynamic distortion or are commonly employed by physicians in their clinical practice. Moreover, we have computed several Wall Shear Stress-based indices. Those indices can provide regions prone to thromboembolic complications and thrombus formation. To eliminate the biasing of our conclusions, we have tested an idealized geometry of the aorta. Nevertheless, the proposed methodology intends to be a conceptual basis adaptable to the analysis of different valves or particular geometries.

## Materials and methods

**Fig. 1:**
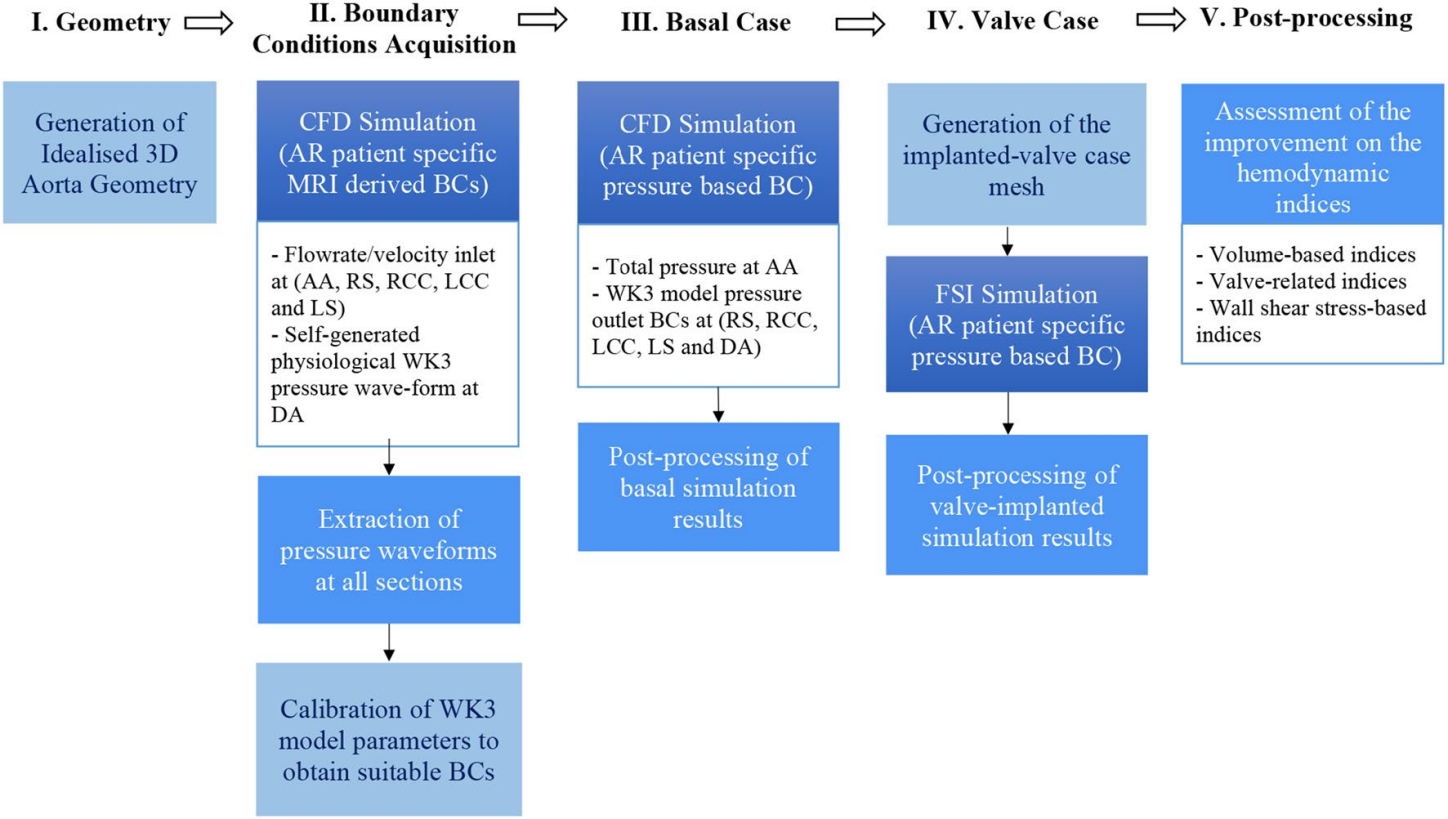
Workflow followed throughout the study. The sections used in the numerical model are: ascending aorta (AA), descending aorta (DA), right subclavian artery (RS), right common carotid artery (RCC), left common carotid artery (LCC), and left subclavian artery (LS)

Figure 1 shows the followed workflow in the present paper. First, the analyzed geometry was generated. Then, moderate-severe AR patient-specific boundary conditions (BCs) (Bolen et al. 2011) were prescribed, and a first CFD simulation was performed. Next, the pressure waveforms at all inlet/outlet sections were extracted and analyzed to find an equivalent description based on a three-element Windkessel model (WK3). This model was calibrated to mimic the underlying behavior of these vessels. A multiple-target optimization routine was created to obtain the right Windkessel parameters for all sections. A second CFD simulation, including pressure BCs, was conducted to check these parameters. Afterward, the valve was introduced on the numerical model, and simulations were performed. From such results, a set of relevant clinical indices were quantified. Finally, we extracted valuable conclusions on the benefits of valve implantation inside the thoracic aorta.

### Geometry description

#### 3D idealized aorta geometry

**Fig. 2:**
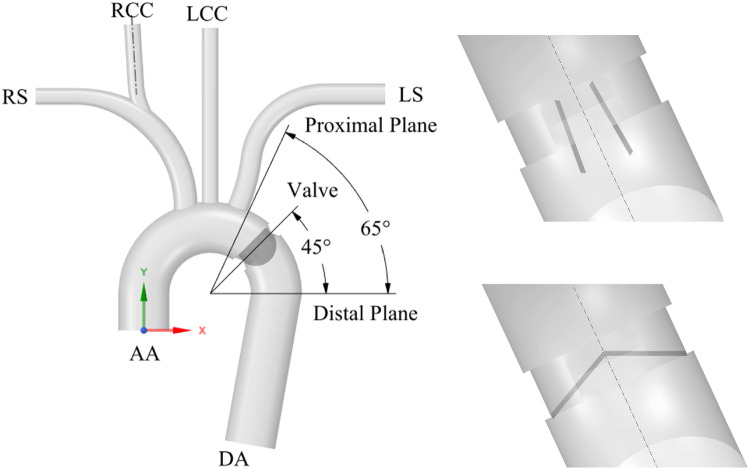
CAD modeling of the idealized aorta geometry: fluid domain overview with sections and valve implantation site with its corresponding proximal and distal planes for pressure gradient assessment (left). Detail of the valve leaflets in its opening (top-right) and closing (bottom-right) positions

To avoid any eventual biasing associated with patient-specific geometrical features, we have employed an idealized aortic geometry (Fig. 2). In this way, we obtain general conclusions on the hemodynamic improvements resulting from valve implantation. The methodology presented in this section can be adapted to analyze a patient-specific aorta. We have created an idealized geometry similar to a previous study (Vasava et al. 2012). We have also left out the coronary arteries as we are keener on analyzing the hemodynamics of greater vessels. We have also avoided the tapering of vessels but for the brachiocephalic trunk, which is divided into two narrower vessels. The exit sections of all supra-aortic vessels were placed at a position sufficiently far from the aortic arc to prevent the appearance of any numerical instabilities (Numata et al. 2016). The geometrical details prescribed in our model (Table 1) were based on healthy male adults (Chang et al. 2020; Manole et al. 2013). From now on, different vessel sections will be referred with Table 1 acronyms, i.e., AA for the ascending aorta section. Full name, i.e., ascending aorta will be written to mention the whole vessel geometry.

**Table 1:** Diameter of all vessel sections

Section	Diameter
Ascending Aorta (AA)	25 mm
Descending Aorta (DA)	25 mm
Brachiocephalic Trunk (BT)	12.1 mm
Right Subclavian (RS)	8 mm
Right Common Carotid (RCC)	8 mm
Left Common Carotid (LCC)	8 mm
Left Subclavian (LS)	10.25 mm

#### Valve geometry

**Fig. 3:**
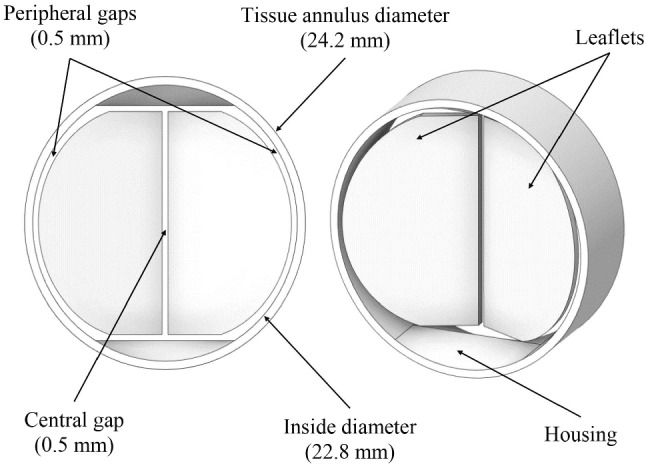
CAD modeling of the bileaflet MHV

A geometry equivalent to the Medtronic Open Pivot ^TM^ AP aortic bileaflet Mechanical Heart Valve (MHV) was selected for the present study (Fig. 3). A suitable valve size (Medtronic PLC 2016) was chosen to fit the aorta diameter at the implantation site (Fig. 2). During systole, the valve leaflets are opened to a position of 5$^{\circ }$ with respect to the valve axial direction (Fig. 2). Blood flows through three channels towards the descending aorta. During diastole, blood forces the leaflets to their closing position following a 60$^{\circ }$ excursion angle (Fig. 2). In this situation, the leakage flow can pass through the peripheral gap ($\sim$50 $\mu$m) and the central gap ($\sim$50 $\mu$m). This leakage volume is critical to account for the regurgitating volume after valve closure at diastole. As the disparity of flow scales (50 $\mu$m - 25 mm) introduced numerical instabilities, we modeled 500 $\mu$m gaps instead of the original ones.

The use of a greater gap distance results in an artificial alteration of leakage volume. A porous sub-model capable of emulating the real 50 $\mu$m gap pressure drop was implemented, to tackle this inconvenience. It allowed us to obtain realistic leakage volumes without compromising the stability of the solution. The valve assigned material was pyrolytic carbon (Medtronic PLC 2016). Each leaflet’s mass and moment of inertia were 0.3022 g and $4.0009\times 10^{-9}$ kg$\cdot$ m$^2$, respectively.

### Numerical simulation of blood flow with the implanted valve

#### Meshing and coupled 2-way FSI

Unstructured tetrahedral elements conformed to the 3D meshes generated in ANSYS $\copyright$ Meshing. A fine grid resolution was needed close to both vessels and valve walls to resolve the laminar viscous sub-layer. The y$^+$ values were kept below 1 at all walls to correctly solve this flow feature (Ansys Inc 2019). The basal case was meshed with a single cell zone, whereas the valve case required a very thorough approach. In such a case, it is necessary to consider the large deformations in the mesh due to motion of the leaflets. Furthermore, the arrangement of fine cells next to leaflets surfaces could hinder the applicability of any remeshing or smoothing techniques to deal with moving meshes. For these reasons, the valve case was meshed following the Chimera technique or overset grid method (Ansys Inc 2019). This method is a fixed grid algorithm where background and component fluid meshes are considered instead of a single mesh. The background mesh remains unchanged throughout the simulation. It is similar to the basal case but for including the valve housing geometry (Medtronic PLC 2016). The component meshes were created as two identical unstructured tetrahedral meshes enclosing each leaflet, with inflation layers stacked on their walls.

The leaflets were deemed to move rotating rigidly around their axes. An Arbitrary Lagrangian-Eulerian (ALE) approach was followed to model the 2-way FSI. Fluid forces and torques and leaflets kinematic properties were employed by ANSYS $\copyright$ Fluent 2019R3 Six-DOF (Degrees Of Freedom) solver to compute the motion of the leaflets. The angular acceleration of each component mesh $\dot{\mathbf {\omega }}_{L}$ is computed as:

1 $$\begin{aligned} \dot{\mathbf {\omega }}_{L}=L^{-1}\left( \sum \textbf{M}_{L}-\mathbf {\omega }_{L}\times L\mathbf {\omega }_{L}\right) , \end{aligned}$$

where *L* is the inertia tensor, $\textbf{M}_{L}$ represents the moment vector of the leaflet, and $\mathbf {\omega }_{L}$ is the angular velocity vector.

Regarding the motion of the leaflets, the fluid/fluid interface, where background and component meshes overlap, changes continuously. We ensured a valid data interpolation by applying similar element sizes on both sides of that interface. This fact and the disparity of scales (sect. 2.1.2) prevented us from reducing the gap to 50 $\mu$m, as an immeasurable cell number would be reached on all meshes due to the cell refinement in the peripheral and central gap regions.

Regarding the data transfer of loads and displacements at the fluid/solid interfaces, a closely coupled approach was pursued. It was set a maximum of 20 coupling iterations per time step. Within each coupling iteration, two inner iterations were first run. Afterward, the Six-DOF solver computed loads on leaflet walls and moved the component meshes according to the obtained displacements. Motion convergence criterion (residuals < 10$^{-3}$) was always achieved before reaching the maximum 20 coupling iterations.

Table 2 summarizes mesh quality parameters and cell size. As part of a mesh sensitivity analysis, we verified that the relative variations of flowrate through each vessel were below 1% after duplicating the number of elements by halving the cell size.

**Table 2:** Summary of mesh size and quality parameters

Data	Basal case	Valve case		
		Background	Component	Overset
No. cells	607586	4367320	2390932	9149184
Max. cell skewness [-]	0.79	0.79	0.79	0.79
Min. orthogonal quality [-]	0.20	0.20	0.21	0.21
Max. aspect ratio [-]	45	29	29	29

#### Solver

Blood flow was simulated through the computation of the incompressible and unsteady RANS equations (Versteeg and Malalasekera 2007) by commercial software ANSYS $\copyright$ Fluent 2019R3. The pressure-based Coupled solver for pressure-velocity coupling was utilized because overset meshes are only supported with this scheme (Ansys Inc 2019). Gradients computation on cell centers were obtained, respectively, through the Least-Squares cell based scheme. In addition, the spatial discretization of momentum equations was performed according to second-order upwind scheme, while the pressure equation followed the second-order approximation. A first order implicit method was selected as transient formulation. Blood was modeled as an incompressible Newtonian fluid with density $\rho =1060\,\hbox {kg}/\hbox {m}^{3}$ and dynamic viscosity $\mu =3.5\, \times \, 10^{-3}\,\hbox {Pa}\cdot \hbox {s}$. This approach can be accepted as shear rates are sufficiently high ($\dot{\lambda } \sim 10^{2}\,\hbox {s}^{-1}$) (Merrill and Pelletier 1967; Chien 1970). Aorta walls, valve leaflets, and housing are considered rigid.

Convergence criteria were: residual levels lower than $10^{-5}$ for all equations and flow rate changes under 0.1% between inner iterations at each section. An adaptive time-stepping method was implemented for the whole cardiac cycle (0.665 s). Time step size is safely increased up to 2.5 ms if the simulation keeps converging for at least ten consecutive time steps. Conversely, if it fails to converge after 50 inner iterations within a single time step, the step size is divided by two until a minimum (0.3125 ms) is reached. This minimum is fixed to facilitate numerical stability when the FSI is enabled and motion of the leaflets is allowed.

Regarding flow modeling, the presence of the valve generates turbulence, and Reynolds number can peak up to 1.07 $\cdot$ $10^{4}$ at AA during systole. Thereby, the $k-\omega$ SST turbulence model (Menter et al. 2006; Wilcox 2006) was selected to obtain a precise force distribution on the leaflets and, as a result, its motion.

#### Implementation of porous sub-model to reproduce valve leakage

A porous sub-model was applied to the simulated valve gaps (500 $\upmu$m) to mimic the leakage flow behaviour from actual valve gaps dimensions (50 μm) during the closure phase. This aspect is deeply relevant to successfully compute different hemodynamic indices (Sect. 2.4). The procedure comprises two phases: the acquisition of the original 50 μm gap resistance curve and its use to calibrate the porous sub-model introduced in the simulated gap (500 μm).

The resistance curve is obtained through an additional simulation as follows: (i) a valve on its closure position is placed in the middle section of a straight tube and respecting all real dimensions, (ii) the geometry was meshed so that ten cells of 5 μm were placed in the gap region, (iii) steady simulations employing the $k-\omega$ SST turbulence model were conducted for different imposed flow rates, (iv) the viscous, $C_{1}$, and the inertial, $C_{2}$, resistance curve coefficients were obtained by fitting the pressure drop vs velocity results.

In ANSYS Fluent, the porous model was deployed by introducing a Source term, $\hbox {S}_i$, which acts as a momentum sink. Its magnitude was calculated through the expression:

2 $$\begin{aligned} S_{i}=-\frac{1}{t_{i}}\left( C_{1}u_{i}+\frac{C_{2}\rho |u_{i}|u_{i}}{2} \right) , \end{aligned}$$

where $C_1$ and $C_2$ are the coefficients mentioned above, $t_i$ is the porous media thickness, and $\hbox {u}_i$ is the speed along the *i*-axis. The sources were introduced on both X and Y momentum equations as their directions were co-planar to the axis that traverses the valve in our layout. The model was activated as soon as both leaflets reached full closure position. Due to numerical instabilities, resulting from sudden flow stoppage, all fluid regions were first patched with zero velocity. This resulted on almost zero flow rates and finite pressure step ups at all sections for the first time steps after porous media is activated. However, this spurious effect does not introduce large deviations on results as the flow rates turn back to their respective values later on and the leakage flow ramps up until it reaches a quasi-stationary level. In this sense, the maximum error would mean only a 0.3% greater leakage volume over the entire cardiac cycle. Conversely, the patching of fluid regions was not needed when the porous zone was deactivated at the beginning of the opening phase, as it is numerically less aggressive.

### Boundary conditions

Usually, aortic valve FSI simulations impose the flow rate previously obtained through experiments as BCs (Dumont et al. 2007; Nobili et al. 2008; Spühler et al. 2018). In our case, the flow rate is the variable of interest. It must be obtained as a result of valve actuation. Therefore, it cannot be prescribed as BC, and all BCs must inevitably be pressure-based rather than flow rate/velocity-based. In this section we explain how the appropriate BCs are obtained according to the adopted workflow (Fig. 1).

#### Basal case pressure waveforms acquisition

We took the flow rate through AA and DA from MRI measurements available (Bolen et al. 2011) on a moderate-severe AR case, as a starting point. Then, we assumed the remaining flow rate was evenly distributed (7.5% of total AA flow rate (Middleman 1972; Benim et al. 2011)) through RS, RCC, LCC, and LS (Middleman 1972; Benim et al. 2011). Moreover, the flow rate corresponding to coronary arteries was directly assigned to DA, increasing up to 70% of the flow through AA (Bolen et al. 2011).

If these flow rates were prescribed as BCs on a CFD simulation, they would lead to physiologically unrealistic pressure waveforms, in terms of shape and magnitude. Furthermore, pressure curves are required in the forthcoming valve simulation. Therefore, we have employed a three-element Windkessel model (WK3) (Westerhof et al. 2009; Vlachopoulos et al. 2011) to produce a realistic pressure-waveform at DA. This pressure-waveform was imposed as BC while keeping the rest of sections as velocity inlet BCs. This way, this first simulation provided us with physiologically realistic pressure waveforms on the remaining sections while keeping the original flow curves (Bolen et al. 2011).

The process to adjust the WK3 model at DA starts by assigning initial values to the parameters, as follows: (i) the compliance *C* and the total resistance $R_t$ (1 ml/mmHg and 1.125 mmHg s/ml) were adopted from the data obtained in the thoracic aorta of usual AR patients (Slordahl et al. 1994), (ii) the characteristic impedance $Z_c$ was estimated as a fixed fraction of 5.6 % $R_t$ (Laskey et al. 1990; Suh et al. 2011) and (iii) the peripheral resistance $R_p=R_t-Z_c$. As the original DA flow curve is discretized temporally, the pressure values (*P*) for every time step (i) are obtained substituting WK3 model-parameters and known flow rate (*Q*) values in the following equation:

3 $$\begin{aligned} P_{i}= & {} \left\{ P_{i-1}\cdot \frac{C\cdot R_{p}}{\varDelta t}+R_{p}\right. \nonumber \\{} & {} \left. \cdot \left[ Q_{i}\cdot \left( 1+\frac{Z_{c}}{R_{p}}+\frac{C\cdot Z_{c}}{\varDelta t}\right) -Q_{i-1}\cdot \frac{C\cdot Z_{c}}{\varDelta t}\right] \right\} \nonumber \\{} & {} \cdot \left\{ 1+\frac{C\cdot R_{p}}{\varDelta t}\right\} ^{-1} , \end{aligned}$$

where $P_{i-1}$, i.e., the pressure value at the previous time step (i-1), is also required. A function programmed in MATLAB $\copyright$ was implemented to get the pressure curve. This curve was set as a pressure outlet BC for the DA section within each time step in ANSYS $\copyright$ Fluent 2019R3 (an User Defined Function was employed).

As mentioned, if a valve is inserted, the flow is modified significantly at outlet sections. For that case, we have assumed an unaltered stagnation pressure waveform at AA resulting from the first basal simulation (Fig. 1). Nevertheless, the valve actuation allows instantaneous static and dynamic pressure variations at AA. This assumption may result in an overestimation of stagnation pressure as the closing valve produces a stagnation pressure loss in retrograde flow conditions. However, in the worst scenario, the stagnation pressure drop through AA is of the orders of magnitude of the dynamic pressure, representing as much as the 0.55% of stagnation pressure during diastole. From this first basal simulation, the resulting stagnation pressure waveform at AA remained unchanged as pressure inlet BC for the rest of our study.

Turbulence intensity level was fixed to a 5% (Ansys Inc 2019) at all inlet/outlet sections, prescribing a turbulent length scale equivalent to the diameter of the vessel. On the other hand, aorta walls were assigned a non-slip boundary condition.

Moreover, the valve occlusion also distorts both pressure and flow rate through DA, RS, RCC, LCC, and LS, preventing the prescription of a hypothetical unalterable static pressure waveform in those sections. Nevertheless, there is an underlying flow-static pressure relationship at all vessels that can be mimicked through the implementation of WK3 model BCs at every section.

This is a satisfactory solution for the numerical analyses that lack available data to impose BC (especially when invasive techniques are involved) (Bonfanti et al. 2019; Karmonik et al. 2014; van Bakel et al. 2018; Pirola et al. 2017). In this study, we took the resulting pressure waveforms at RS, RCC, LCC, LS, and DA from the first CFD simulation, and developed a multi-objective optimization algorithm in MATLAB $\copyright$ to obtain the corresponding Windkessel parameters for each outlet section.

#### Multi-objective optimization algorithm for WK3 model parameters estimation

The algorithm searches the WK3 parameters within the physiological range (Slordahl et al. 1994; Razzolini et al. 1994) that generate the best fitting pressure waveforms to the ones from first simulation. It minimizes the euclidean norm of the relative error

4 $$\begin{aligned} \Vert e_{R} \Vert =\sqrt{\frac{\sum _{i=1}^{N_{steps}}|P_{g}(i)-P_{t}(i)|^2}{\sum _{i=1}^{N_{steps}}|P_{t}(i)|^2}} , \end{aligned}$$

between the target $P_t$ and generated $P_g$ pressure waveforms, respectively.

First, the algorithm computed $R_{t}$ as the ratio between the mean pressure and flow rate from the basal case for each outlet. Then, different combinations for *C* and $Z_{c}/R_{t}$ were computed until the global minimum for $\Vert e_{R} \Vert$ was found with the constraint equation ($R_p=R_t-Z_c$). Next, a new set of parameters in the previous ones’ neighbourhood was tested. The obtained parameters (Table 3) were employed to generate pressure profiles for each boundary. A second simulation (basal in Fig. 1) employed stagnation pressure waveform as pressure inlet at AA and WK3 model-based pressure outlet BC for the remaining sections (Table 3). The results matched the first basal simulation and will serve as a reference to compare the valve simulation results.

**Table 3:** Best fitting WK3 model parameters resulting from the multi-objective optimization algorithm

Parameter	RS	RCC	LCC	LS	DA
$R_{t}$ [mmHg$\cdot$s/ml]	10.74	10.78	10.74	10.63	1.13
$Z_{c}$ [mmHg$\cdot$s/ml]	0.51	0.50	0.45	0.58	0.06
$R_{p}$ [mmHg$\cdot$s/ml]	10.23	10.28	10.29	10.05	1.07
*C* [ml/mmHg]	0.11	0.10	0.10	0.11	0.97
$\Vert e_{R} \Vert$ [%]	2.46	2.92	3.19	1.63	0.38

### Hemodynamic indices

In this section, we use various indices to assess the viability of the proposed technique. A first group quantifies several blood volumes and is commonly employed by physicians in their clinical practice: The Stroke Volume (SV), the Regurgitant Volume (RVol), and the Regurgitant Fraction (RF) (Solomon 2007). SV is the volume of blood pumped out of LV during the systole, RVol is the volume flowing back to LV during diastole, and RF is the ratio between Rvol and SV. Aortic regurgitation severity can be assessed by classifying RVol and RF employing Doppler Echocardiography (DE) or Cardiovascular Magnetic Resonance (CMR) (Gelfand et al. 2006) (Table 4).

**Table 4:** Assessment of aortic regurgitation severity

Index	Mild	Moderate	Severe	Technique
RVol [ml/beat]	<30	30-59	$\ge$60	DE (Solomon 2007)
RF[%]	$\le$15	16-27	>27	CMR (Gelfand et al. 2006)

The flow and hemodynamic distortion introduced by the implanted valve must also be considered for its mechanical design. It can be estimated through two parameters: the Transvalvular Pressure Gradient (TPG) and the Effective Orifice Area (EOA) (Bronzino 1999). TPG is the pressure gradient between proximal and distal valve section planes depicted in Fig. 2 during systole. Distal plane has been placed where the velocity profile becomes more uniform and the pressure is recovered. Proximal plane was located slightly distal to LS root to avoid LS influence. Moreover, EOA is computed as

5 $$\begin{aligned} \textrm{EOA}=\frac{Q_\textrm{rms}}{51.6 \sqrt{\varDelta \overline{P}}} , \end{aligned}$$

where $Q_{rms}$ is the root mean square of systolic/diastolic flow rate and $\varDelta \overline{P}$ the mean systolic pressure drop. EOA, $Q_{rms}$ and $\varDelta \overline{P}$ are usually expressed in cm$^2$, cm$^{3}$/s and mmHg, respectively.

Finally, the long-term implantation of MHVs can ease the appearance of local regions of blood where blood stasis, platelets activation, or hemolysis may represent a major concern. Wall shear stress (WSS) computation can be employed to obtain several indices. They can help distinguishing and classifying the likelihood of these shortcomings: Time Averaged Wall Shear Stress (TAWSS), Oscillatory Shear Index (OSI), Endothelial cell activation potential (ECAP), and Relative Residence time (RRT). TAWSS, computed through the valve closure phase, is quite important to address the stresses on all valve gaps. OSI is defined as

6 $$\begin{aligned} \textrm{OSI}=\frac{1}{2} \left( 1-\frac{| \int _{0}^{T} \textrm{WSS} \quad dt)|}{\int _{0}^{T} |\textrm{WSS}| \quad dt}\right) , \end{aligned}$$

and is capable of identifying critical flow oscillations during the cardiac cycle. ECAP is the ratio between OSI and TAWSS. It is useful for identifying regions prone to develop intraluminal thrombus or abdominal aneurysm (low TAWSS and large OSI simultaneously). RRT is the residence time of blood particles. It is a helpful indicator of the likelihood of platelet aggregation in the endothelium and can be obtained from the equation:

7 $$\begin{aligned} \textrm{RRT}=\frac{1}{\textrm{TAWSS}(1-2 \cdot \textrm{OSI})} \end{aligned}$$

## Results

### Comparison between basal and valve-implanted cases

**Fig. 4:**
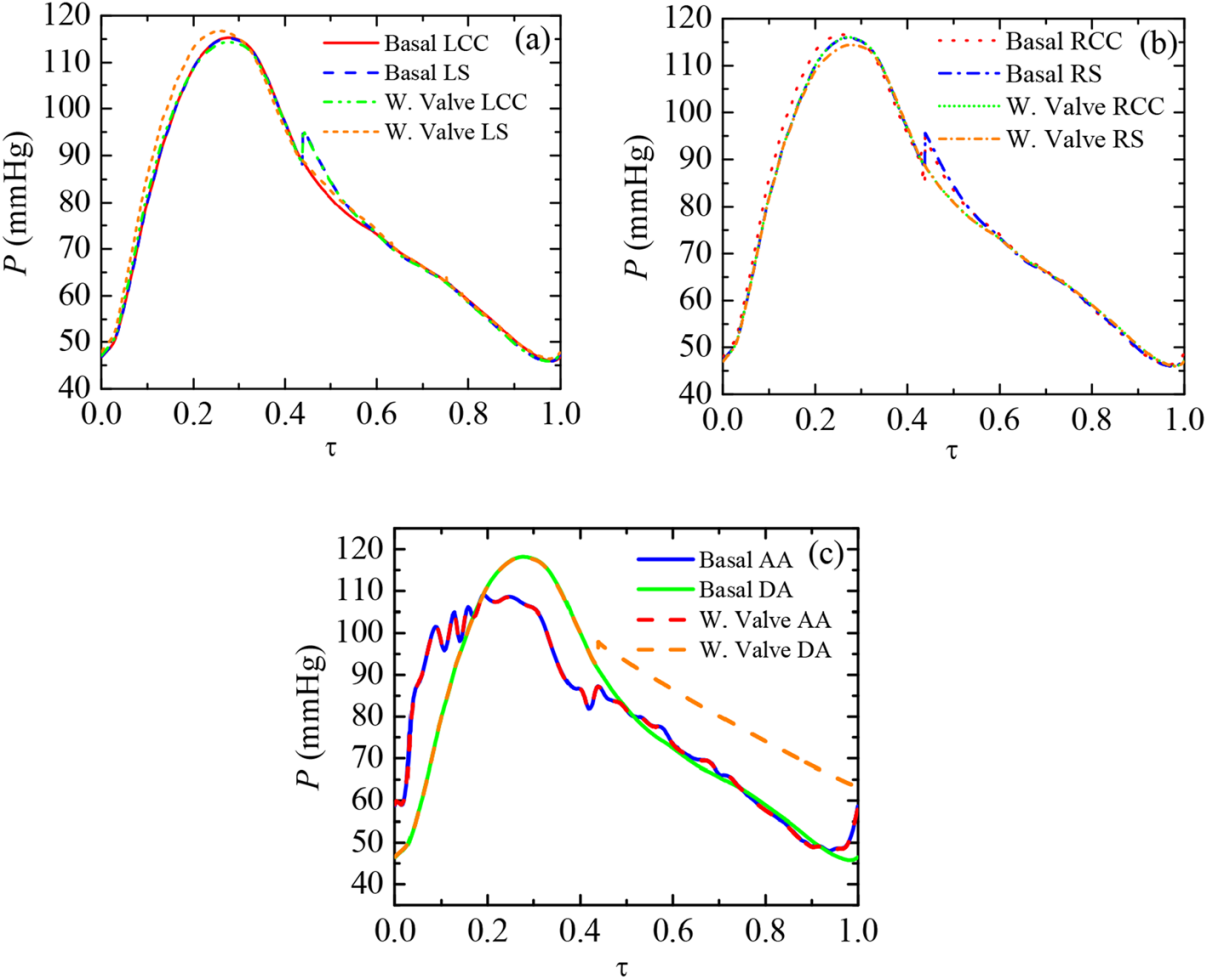
Comparison of pressure distribution *P* with dimensionless time $\tau$ over the entire cardiac cycle (basal case and valve implanted case). Small vessels: LCC and LS **a** and RCC and RS **b**. Aorta: AA and DA **c**

During diastole ($0.37 \le \tau \le 1$, where $\tau$ is the dimensionless time and the cardiac cycle is the characteristic time), the valve closure leads to a sudden increase in pressure at all vessels due to flow stoppage introduced numerically by porous media (Fig. 4). However, RS, RCC, LCC, and LS pressure levels are rapidly restored to their respective basal values as the diastolic phase advances (Fig. 4a and b). The regurgitation through those vessels becomes virtually unaltered (Rose et al. 1954) as there is no occlusion on them. Conversely, pressure at DA becomes higher than its basal counterpart right until the end of diastole (Fig. 4c). The blockage generates a great pressure drop across the valve due to the narrow passages through both central and peripheral gaps. DA pressure in valve case is 6.51 mmHg higher than the basal case right from the beginning of the closure phase ($\tau =0.44$). It slowly decays afterwards until the end of diastole ($\tau =1$), where pressure at DA ends being 16.69 mmHg higher than basal.

**Fig. 5:**
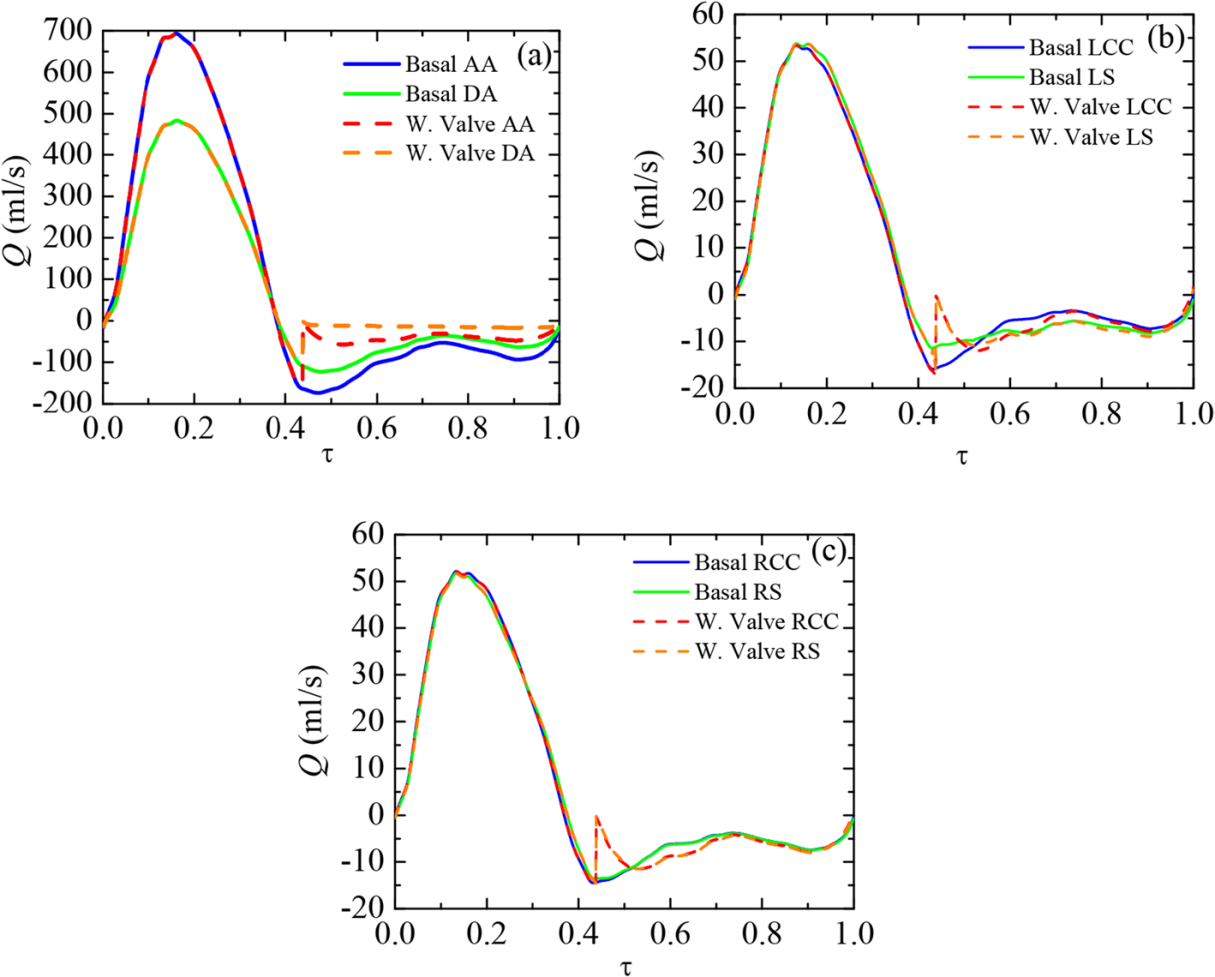
Comparison of flow distribution *Q* with dimensionless time $\tau$ over the entire cardiac cycle (basal case and valve implanted case). Aorta: AA and DA **a**. Small vessels: LCC and LS **b** and RCC and RS **c**

Regarding the flow rate, the valve occlusion effect in the aorta is noticeable (Fig. 5a). The time average regurgitant flow rate through DA from valve closure to the end of diastole ($0.44 \le \tau \le 1$) drops down about five times owing to the valve (from 68.20 ml/s to 13.98 ml/s). Likewise, during the same period, AA time average flow rate is reduced by more than a half (from 96.7 ml/s to 40.95 ml/s). Furthermore, RS, RCC, LCC, and LS flow distributions are slightly modified (<1% over the entire cardiac cycle) (Fig. 5b and c). Just the spurious local stoppage when the porous media model is activated.

### Resulting valve motion profile

**Fig. 6:**
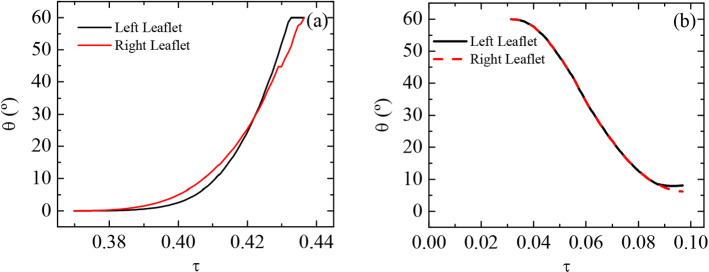
Evolution of leaflets excursion angles during **a** closure and **b** reopening phases

At the onset of regurgitation ($\tau =0.37$), blood exerts a torque on the two leaflets, forcing them to deflect towards their closing position (Fig. 6a). Such torque and the leaflets rotation velocity increase as their frontal surface becomes more exposed to flow. The closing phase is quite similar for both leaflets (Fig. 6a). Slight differences can be attributed to the oscillations induced by the turbulence in such valve arrangement (left and right leaflets closure times are 43.8 and 44.4 ms, respectively).

Figure 6b shows the reopening phase. The flow accelerates at systole onset, and both leaflets start to move back to their original open position. Valve leaflets reopen to about $7^{\circ }$ due to the interaction between the opened three channels. The unstable nature of pulsatile flow introduces some oscillations on the leaflets, making them turn back and forth during systole as blood passes through the three open channels. These oscillations do not alter the flow rate, as blood flow is not occluded at all. Since we are keener on understanding the regurgitation reduction due to valve actuation during diastole, we discarded flow quantification during the re-opening phase, where leaflets are left free to move.

### Analysis of valve-related indices

**Fig. 7:**
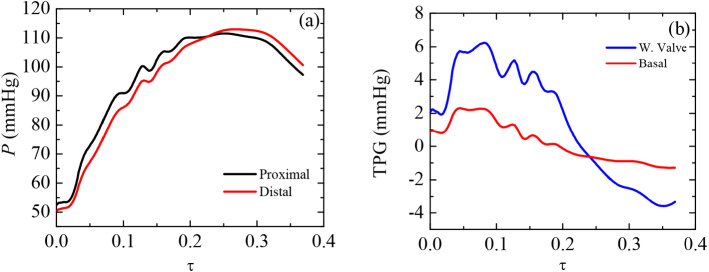
Proximal and distal pressures computed to obtain TPG for the valve case (left). TPG comparison against pressure drop for the already defined proximal and distal sections on the basal and valve cases (right). Recordings were obtained at systole ($0 \le \tau \le 0.37$)

Figure 7a shows the proximal and distal pressures employed to compute TPG during systole ($0 \le \tau \le 0.37$). Both pressures show a parallel trend until the systolic peak is reached $(\tau =0.15)$. Then, flow decelerates, and both pressures start getting closer until they match at $(\tau =0.225)$. Immediately after, distal pressure becomes larger than proximal during the remaining systole. TPG is compared against the basal case pressure drop calculated in the same locations as in the valve case (Fig. 7b). As expected, the valve, despite being in its opening position, introduces an additional pressure gradient that peaks in the period ($0.05 \lesssim \tau \lesssim 0.17$). This maximum additional pressure drop is 4 mmHg, i.e., twice the basal pressure drop in the most adverse period. We understand that this pressure loss may be slightly higher in a real valve when larger leaflets needed to reduce the gap to 50 $\mu$m would have been considered. Nevertheless, the largest amount of pressure drop is due to luminal area contraction ($A_\textrm{valve}/A_\textrm{basal}=0.83$) resulting from housing geometry (Sec. 2.1.2).

Other relevant parameters related to the pressure loss introduced by the valve are the mean TPG, the TPG calculated taking the maximum pressures at the considered sections ($\hbox {TPG}_{\mathrm {peak-to-peak}}$), the maximum instantaneous TPG ($\hbox {TPG}_{\mathrm {max-inst}}$), the root mean square of flow rate through DA ($\hbox {Q}_{\mathrm {RMS-sys}}$) and the EOA. In our case, the values obtained during the systolic period ($T_{\textrm{sys}}=0.245$ s) were: $\overline{\textrm{TPG}}=1.50$ mmHg, $\hbox {TPG}_{\mathrm {peak-to-peak}}=-1.46$ mmHg, $\hbox {TPG}_{\mathrm {max-inst}}=6.24$ mmHg, $\hbox {Q}_{\mathrm {RMS-sys}}=339.05$ cm$^3/$s and EOA$=5.36$ cm$^2$. These values indicate a well-positioned valve that does not obstruct flow (Bronzino 1999).

**Fig. 8:**
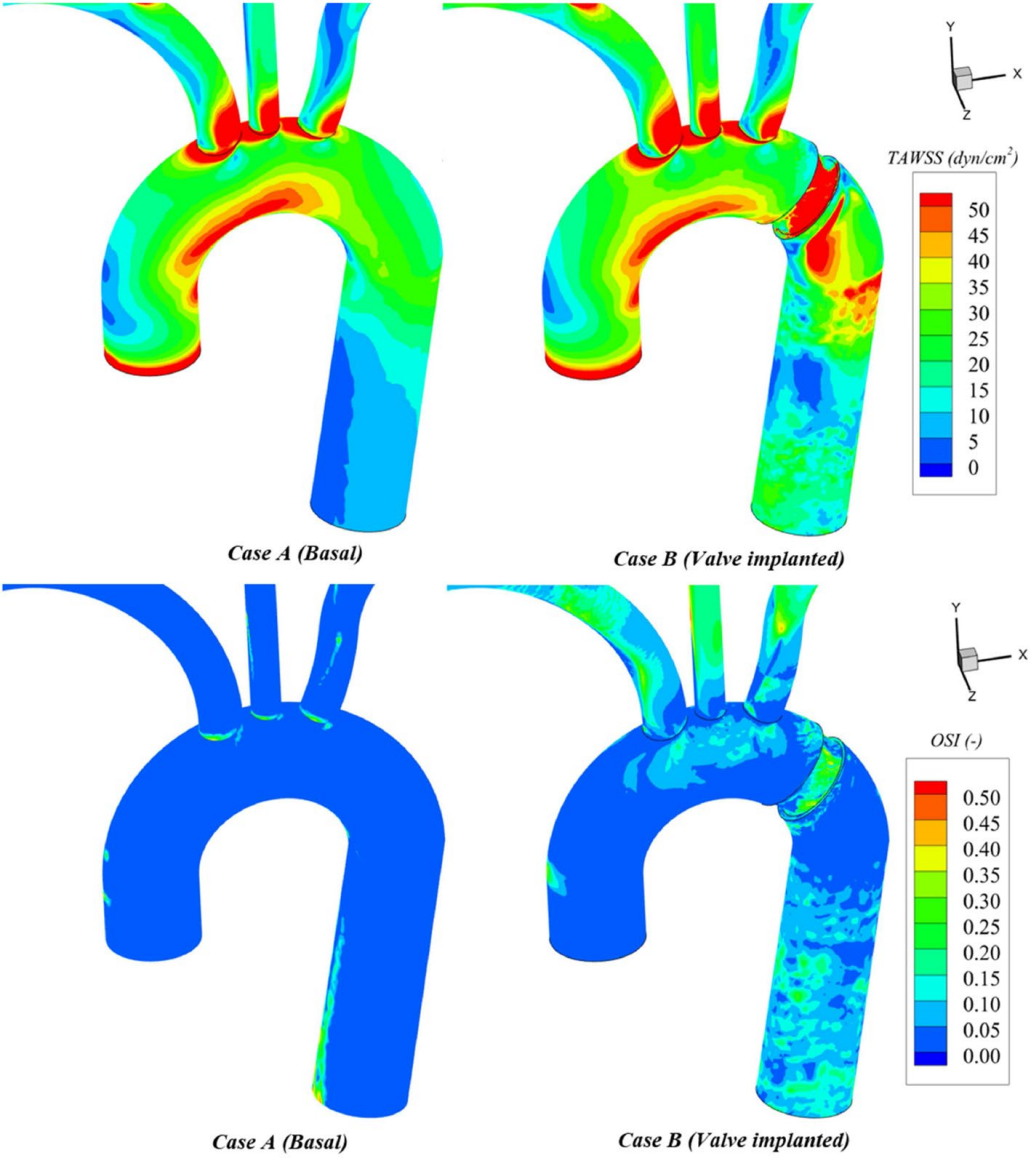
TAWSS (top) and OSI (bottom) comparisons: basal case (left) and valve case (right)

Regarding WSS-indices, TAWSS contours (Fig. 8) show an almost unchanged pattern in the ascending aorta and the vicinity of the valve. A general increase is present in the descending aorta. This increase is more evident in the surroundings of valve housing due to the presence of large velocity jets flowing through the valve gaps during the closure phase. Compared to the lower values from the basal case, the general increase of TAWSS to levels over 15 dyn/cm$^{2}$ found on regions distally to the valve, is quite satisfactory. Low shear stresses (< 4 dyn/cm$^{2}$) stimulate an atherogenic phenotype, whilst higher ones (> 15 dyn/cm$^{2}$) induce endothelial quiescence and an atheroprotective gene expression profile (Malek et al. 1999).

The issuing of jets increases TAWSS locally and the oscillatory pattern throughout the cycle in the aortic walls. Figure 8 shows an augmentation of OSI values up to 0.20 in regions placed distally to the valve. OSI becomes higher in supra-aortic vessels as the flow is suddenly stopped and then compensated by a local increase in regurgitation.

**Fig. 9:**
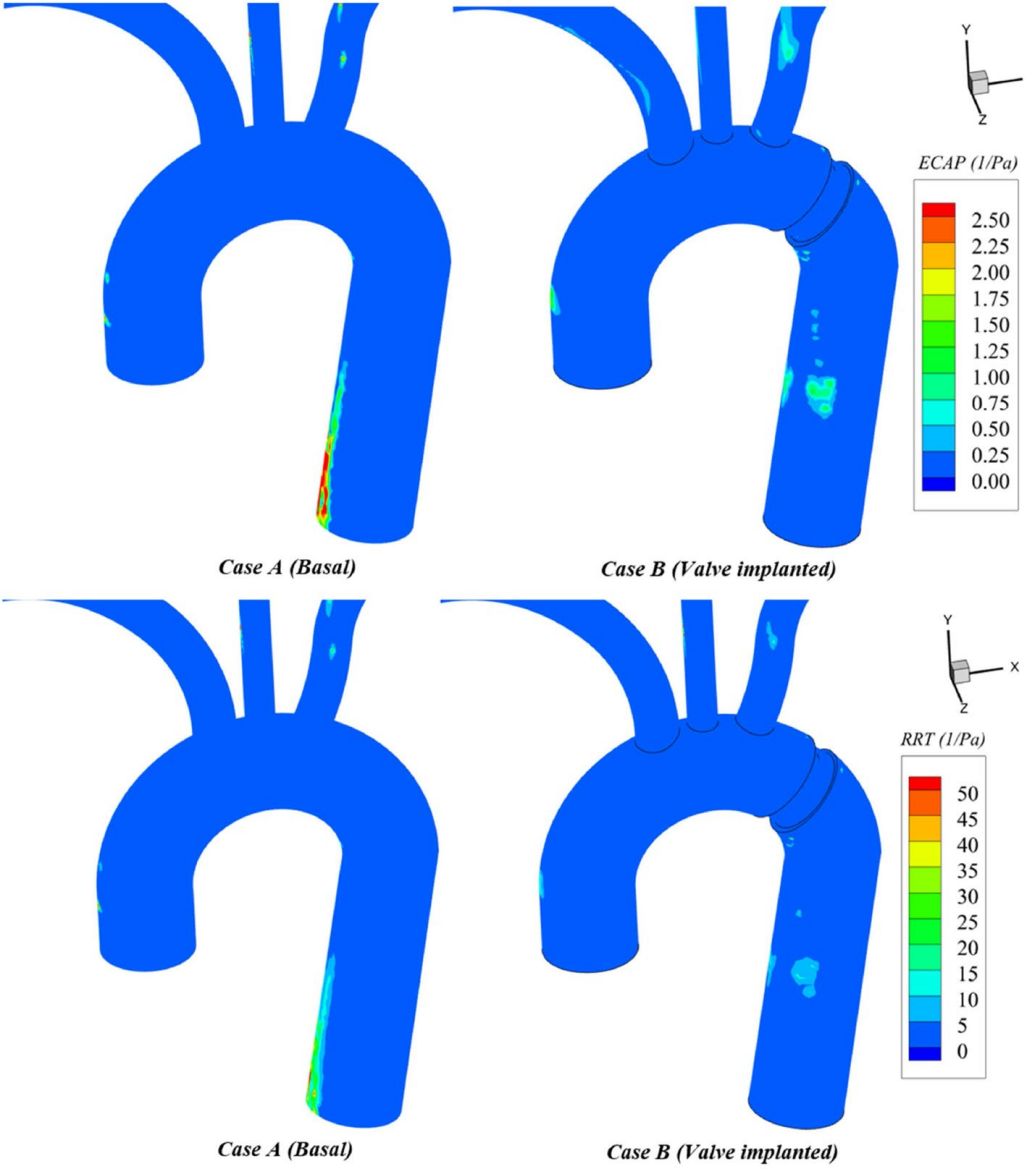
ECAP (top) and RRT (bottom) comparisons: basal case (left) and valve case (right)

The presence of the valve does not substantially alter ECAP values (Fig. 9). Distally to the valve, the appearance of small regions subjected to an ECAP over 1 $\text{ Pa}^{-1}$ is due to the general increase of OSI caused by the accelerated blood profile on the walls resulting from the jet-like peripheral gaps leakage. ECAP is reduced at the inner side of the descending aorta due to the compensated increase of TAWSS and OSI. This reduction is significant compared to the basal case, where larger ECAP values appear because higher OSI and smaller TAWSS levels ($< 5\quad \text{ dyn/cm}^{2}$) coexist. Finally, RRT values are reduced in valve case due to the washing out of descending aorta walls eased by the peripheral leakage. However, the levels remain unchanged for the rest of the aortic walls.

## Discussion

The results obtained, yet representing the outcome of a conceptual approach, can characterize both the strengths and weaknesses of Hufnagel’s approach. The strengths imply a substantial reduction of regurgitant volume (RVol) flowing back towards LV, which is the primary objective of valve implantation for AR cases. Conversely, the weaknesses can be thought of as the uncontrolled appearance of flow areas prone to thrombus formation, platelet activation, hemolysis, and platelet aggregation in the endothelium at the vicinity of the valve.

### Improvements on regurgitant volume (RVol) and regurgitant fraction (RF)

Section 3 results show a significant regurgitant flow reduction due to the occlusion in descending aorta. Computed RVol is reduced by a remarkable 52% (from 39.64 ml/beat to 19.03 ml/beat) as a result of valve implantation in just a single cardiac cycle. The considered case would change from being a moderate to a mild regurgitation scenario (Table 4).

Additional data can be extracted from the analysis of the regurgitating volume coming from the descending aorta. There are two contributions to this particular volume during diastole when MHVs are employed (Bronzino 1999): the closing volume (related to the closure period when the valve is moving) and the leakage volume (related to the remaining of diastole)(Bronzino 1999; Siedlecki 2018). In our case, the closing volume is 2.24 ml/beat (29%), and the leakage one is 5.39 ml/beat (71%). The analysis of the available data (Bronzino 1999) shows more balanced numbers (59% and 41% for the closing and leakage volumes, respectively). Nevertheless, these data are only illustrative as they depend on the patient and the valve. Be it as it may, the leakage volume we obtained is inherently larger due to porous media application, leaving us in a more adverse scenario.

Regarding RF, the partial occlusion of the valve at the beginning of the following systolic phase does not produce substantial changes, and SV remains the same (105.65 ml/beat). Therefore RF is reduced from an original 37.52% to 18.01%. According to CMR technique assessment (Table 4), the case would pass from being graded as a severe one to a moderate one and very close to the mild AR grading threshold.

### WSS-related risks

Section 3 results indicate that the flow is experiencing greater directional changes due to valve action. OSI values are increased up to 0.2 in some regions of the descending aorta (OSI>0.1 values are considered high (Williams et al. 2010)) (Fig. 8). Nevertheless, as TAWSS levels from this region are increased to 15 dyn/cm$^2$, the risk of developing atherosclerotic plaques is contained (Malek et al. 1999; Ku et al. 1985; Frydrychowicz et al. 2009) (Fig. 8). This is confirmed by the virtually unaltered ECAP index (Fig. 9). Likewise, the endothelium does not present platelet aggregation risk since residence times remain unchanged (Fig. 9).

**Fig. 10:**
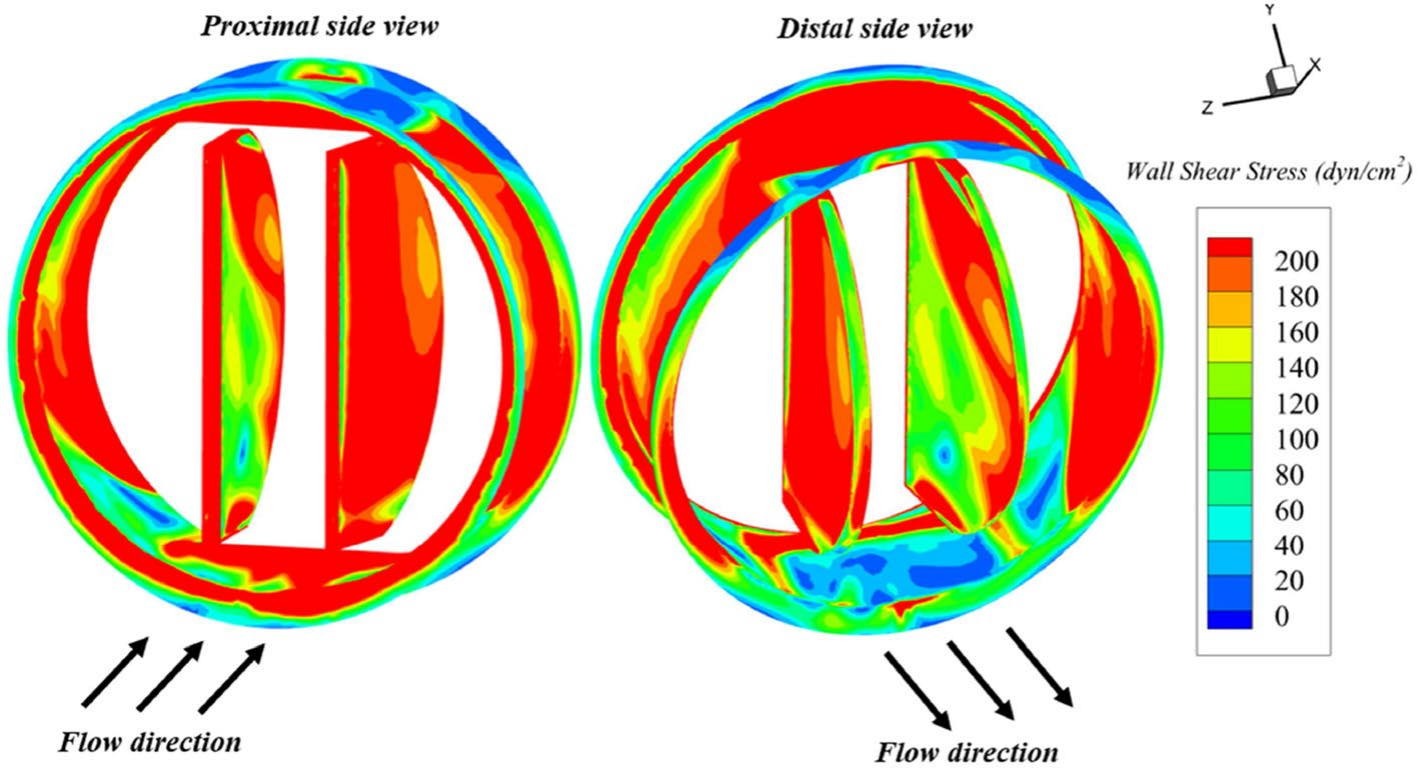
WSS values over the valve surfaces at peak systole ($\tau =0.15$)

Besides the information provided by WSS-related indices, additional phenomena associated with high velocities must be considered in valve design. Figure 10 shows the instantaneous WSS over the valve surfaces at the systolic peak $\tau =0.15$. Extreme WSS values appear at both proximal and distal (200 dyn/cm$^2$) sides of the valve. The housing edges, slants, and the external leaflet faces located at the proximal side concentrate the highest values. The lowest values are found at distal housing slants and edges where the flow is detached.

**Fig. 11:**
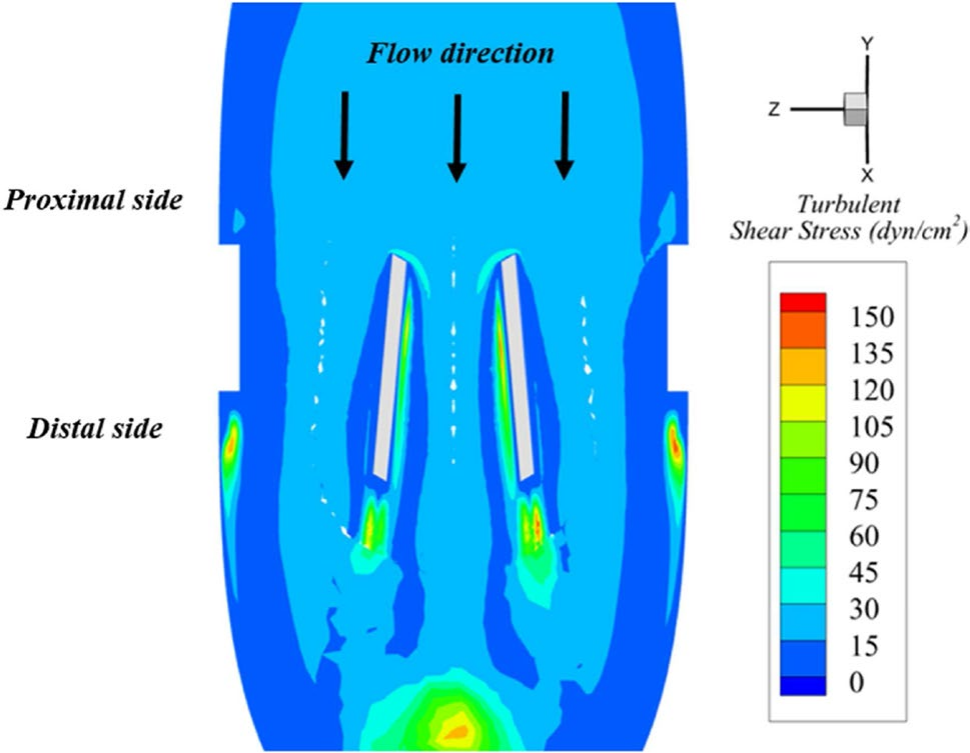
Values of Turbulent Shear Stress (TSS) on a valve longitudinal plane at peak systole ($\tau =0.15$)

Moreover, areas where flow turbulence is more intense are directly associated with thrombosis, thromboembolism, anticoagulation related hemorrhage, thrombus formation, hemolysis, tissue overgrowth, and endothelium damage (Bronzino 1999; Nygaard et al. 1994). Thromboembolic complications and thrombus formation represent about 75% of all valve-related complications for patients with MHV (50% for aortic bioprostheses)(Nygaard et al. 1994). Figure 11 shows the turbulent shear stresses (TSS) distribution on the valve longitudinal plane. TSS are commonly calculated for MHV design and are defined as:

8 $$\begin{aligned} \textrm{TSS}=\nu _{t} \left( \frac{\partial \overline{u}}{\partial y}+\frac{\partial \overline{v}}{\partial x} \right) , \end{aligned}$$

where $\nu _{t}$ is the turbulent viscosity and $\left( \frac{\partial \overline{u}}{\partial y}+\frac{\partial \overline{v}}{\partial x} \right)$ is the strain rate. The only noticeable areas within the turbulence field are the wakes located right at the back of the housing and the leaflet surfaces. Nevertheless, those areas are marginal, and their TSS values (60–150 dyn/cm$^2$ ) do not entail any risk for platelet aggregation, as a continuous shear of 150 dyn/cm$^2$ during 300 s is needed (Bronzino 1999). Despite this, we are aware that intrinsic damage can be fostered due to the valve presence. Leaflets’ multiple journeys can induce cumulative damage that may promote thrombosis and embolization (Nygaard et al. 1994; Brown et al. 1975). Erythrocytes can also be harmed if subjected to shear stresses (10 to 100 dyn/cm$^2$) when foreign surfaces are present (Mohandas et al. 1974).

### Long-term effects of valve implantation

From a technical point of view, performing a simulation to calculate the long-term effects of valve implantation in large vessels is far from being trivial and forbidden computationally. First, detailed LV properties are required to resolve the coupling of LV and aorta. A large number of assumptions, hypotheses, and simplifications must be adopted for the analysis. Likewise, numerical instabilities can make it unfeasible to perform an FSI analysis where motions occur in a time-lapse of milliseconds. This kind of analysis was deemed far from our objectives focused on analyzing the short-term effects of a cardiac cycle valve actuation. Nevertheless, the simulation results can be analyzed from a long-term perspective, as some effects show up from a few weeks to months after the operation (Gaasch et al. 1978).

**Fig. 12:**
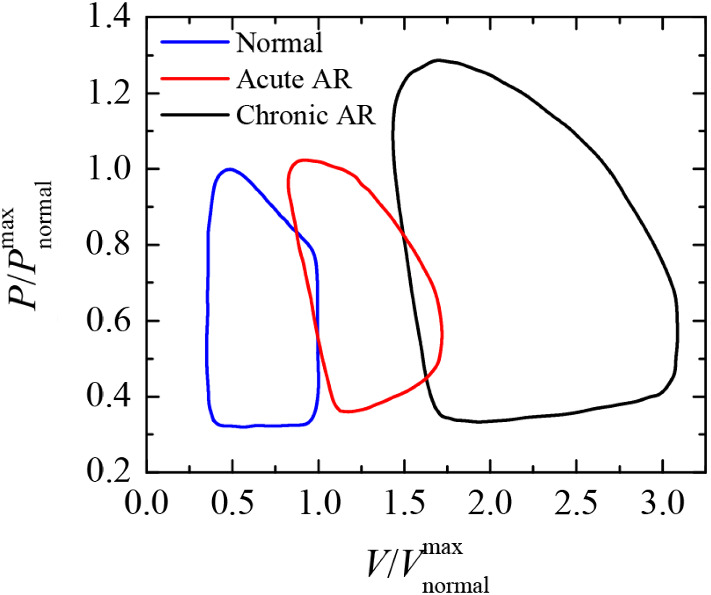
Frank-Starling diagrams of patients with acute severe and chronic severe AR as compared with a normal left ventricle. Data from (Lang et al. 2016) were normalized to the maximum pressures and flow rates measured for a healthy patient, respectively

These are expected to mimic long-term ones seen when SAVR is practiced on patients diagnosed with aortic insufficiency. In this way, following Frank-Starling mechanism (Fig. 12), which describes the coupling of volume and pressure at the LV (Vlachopoulos et al. 2011), the closed-loop curve would be gradually modified to a flatter one. Both end systolic and diastolic volumes would be reduced and also the systolic stress on LV walls. That would be similar to a healthy patient (Lang et al. 2016). Therefore, both preload and afterload would be reduced. Since RVol becomes smaller and hence the LV does not need to eject a greater amount of blood to compensate the subsequent retrograde flow circulation, SV would be reduced too. At the same time, the ejection fraction (EF) would be increased, indicating an improvement of LV function (Rothenburger et al. 2003). This would imply an alleviation of O$_{2}$ consumption, an increase of aorta O$_{2}$ saturation, and a reduction of cardiac output (CO).

Besides, as the end-diastolic volume would become smaller, so it would gradually be the left ventricular dimension at end-diastole (Gaasch et al. 1978; Rothenburger et al. 2003), following a ventricular reverse remodeling process (Seldrum et al. 2019). It is possible that the time required for reaching the same level of amelioration of LV and hemodynamics would tentatively be greater than that resulting from SAVR intervention. However, the benefits of Hufnagel’s technique are extendable to patients disqualified from SAVR or TAVR at the ascending aorta.

### Limitations

The purpose of the present work has been to perform a conceptual approach on the benefits of Hufnagel’s technique in a moderate-severe AR patient employing an ideal geometry. Future works will switch towards a patient-specific paradigm (segmented geometries from CT scan and particular BCs), valve positioning and typing. In like manner, we will gradually avoid simplifications: considering the non-Newtonian behaviour of the blood, the inclusion of coronary arteries, the addition of vessel compliance and keeping the damaged aortic valve to check hemodynamics at the aortic root level. Finally, we aim to compare CFD/FSI results with pure experimental ones to refine the fit between WK3-based waveforms and real ones.

## Conclusions

In the present work, we have numerically evaluated the effect of positioning a bileaflet MHV in the descending aorta distally to the LS artery on a moderate-severe AR case. The reduction on RF from a basal case of 37.52–18.01% within a single cardiac cycle, clearly shows the remarkable efficacy of the proposed technique. Moreover, it is implied that the evaluated RF reduction over one cardiac cycle can be associated with an amelioration of LV function. Ventricular reverse modeling is expected in the long term following the aforementioned conventional procedures.

We have also evaluated the risk of incompatibilities derived from valve implantation in the descending aorta, such as hemolysis, platelet aggregation, and atherogenesis. The values of a complete set of WSS-based indices have shown that those incompatibilities’ risk is minimal. As the flow rate is lower in the descending aorta, that risk would be lower than the one obtained in conventional implantation in the ascending aorta (Bronzino 1999; Nygaard et al. 1994; Brown et al. 1975; Mohandas et al. 1974).

The proposed methodology can provide resources to quantify cases lacking some invasive clinical data. It is adaptable to any particular geometry, and it is designed to provide parameters commonly employed in engineering. Finally, it can also assess clinical parameters that are easily understandable by physicians to account for the improvement introduced.
